# Pesquisando Compostos de Ocorrência Natural para Tratar Hipertensão

**DOI:** 10.36660/abc.20210869

**Published:** 2021-11-22

**Authors:** Diego Santos Souza, Danilo Roman-Campos

**Affiliations:** 1 Universidade Federal de São Paulo Escola Paulista de Medicina Departamento de Biofísica São Paulo SP Brasil Laboratório de CardioBiologia, Departamento de Biofísica, Escola Paulista de Medicina, Universidade Federal de São Paulo, São Paulo, SP – Brasil

**Keywords:** Doenças Cardiovasculares, Hipertensão, Dieta, Produtos Biológicos, Mortalidade, Prevenção e Controle, Sauromatum Gluttatum/uso terapêutico, Ratos

As doenças cardiovasculares (DCs) são a principal causa de morte em todo o mundo. De forma preocupante, aproximadamente 17,9 milhões de pessoas morreram de CDs em 2019, representando quase 32% das mortes globais. Além disso, quase três quartos das mortes por DC ocorrem em países de baixa e média renda, o que é um fator de risco para os brasileiros.^1^ Existem vários mecanismos subjacentes envolvidos nas DCs, e a hipertensão é um contribuidor significativo. Hoje, estima-se que cerca de 1,28 bilhão de adultos com idade entre 30 e 79 anos tenham hipertensão no mundo. Além disso, estima-se que 60–70% da hipertensão em adultos seja atribuída à obesidade, associada à resistência à insulina, dislipidemia e síndrome metabólica.^2^ Além disso, quase 50% dos adultos com hipertensão desconhecem sua condição e apenas 20% têm hipertensão sob controle.^3^

De acordo com o American College of Cardiology e a American Heart Association,^4^ a pressão arterial normal (PA) é considerada quando a pressão arterial sistólica (PAS) é < 120 mm Hg e <80 mm Hg para a pressão arterial diastólica (PAD). A PA elevada é considerada quando a PAS é 120-129 e a PAD é <80 mg. Os estágios 1 e 2 de hipertensão são considerados quando a PAS é 130-139 mm Hg ou > 140 mm Hg, respectivamente, ou PAD é 80-89 mm Hg ou> 90 mm Hg, respectivamente. Esses valores também seguem a diretriz brasileira mais recente para o diagnóstico de hipertensão.^5^

Os números nos mostram que a busca por novos compostos para amenizar a hipertensão é de grande importância para o tratamento de pacientes em todo o mundo. As terapias de reaproveitamento são uma possibilidade. Por exemplo, um estudo piloto recente demonstrou que uma única dose de infliximabe pode reduzir os níveis médios e de PAD imediatamente após sua infusão, em comparação com o placebo em pacientes com hipertensão. No entanto, nem todo paciente pode se beneficiar com esse tipo de terapia.^6^

Nesse cenário, os produtos naturais desempenham um papel fundamental. A implementação de produtos naturais tem aumentado nas últimas décadas. Em um estudo de revisão aprofundado que cobriu produtos totalmente naturais como fontes de novos medicamentos, de 1981 a 2019 aprovado pela Food and Drug Administration, aproximadamente 1946 novos compostos foram aprovados para tratar doenças humanas. No âmbito dos anti-hipertensivos, foram autorizados 82 compostos naturais.^7^ Porém, apesar do número crescente de compostos naturais autorizados, ainda é necessário encontrar novos compostos para o tratamento da hipertensão, uma vez que os pacientes não respondem a uma determinada classe de anti-hipertensivos ou não aderem ao tratamento.

Bibi et al., ^8^ descrevem um novo extrato vegetal bruto de *Sauromatum guttatum* para tratamento experimental da hipertensão. No manuscrito, os autores avaliam o potencial de aplicação terapêutica do extrato vegetal no modelo animal clássico de hipertensão induzida por sal elevado em ratos (ratos DOCA-hipertensos). Seu estudo descobriu que animais hipertensos tratados diariamente por oito semanas com *S. guttatum* atenuaram significativamente a pressão arterial de uma maneira dependente da dose. Além disso, 300 mg/kg de *S. guttatum* tiveram um efeito anti-hipertensivo semelhante ao verapamil 15 mg/kg tratado durante o mesmo período. O uso de anéis aórticos isolados de ratos hipertensos favoreceu o relaxamento dependente da acetilcolina. É importante ressaltar que os anéis aórticos isolados de ratos hipertensos tratados por via oral com o extrato bruto de *S. guttatum*. na dose de 300 mg/kg por 28 dias teve uma potência semelhante sobre o efeito relaxante dependente de acetilcolina contra contrações induzidas por fenilefrina que foi observado nos animais controle. Curiosamente, os anéis aórticos isolados de animais hipertensos tratados com verapamil 15 mg/kg por 28 dias mostraram apenas uma melhora modesta no modelo experimental de hipertensão. Aparentemente, o mecanismo envolvido no efeito de relaxamento do *S. guttatum* inclui a ativação de receptores muscarínicos nas células endoteliais e a estimulação da produção de óxido nítrico. Além disso, o extrato bruto pode interagir com o canal de cálcio do tipo L encontrado nos anéis aórticos, o que também pode contribuir para o efeito anti-hipertensivo observado. Desta forma, estudos futuros devem isolar o principal agente antipertensivo presente no extrato bruto de *S. guttatum* para desvendar o seu potential uso para o tratamento de hipertensão, inclusive em outros modelos experimentais.

Apesar do crescente número de produtos naturais aprovados como agentes terapêuticos, considerando a hipertensão, ainda é necessário encontrar novos compostos que atuem em doses baixas. Além disso, um composto natural pode ser útil para pacientes que não respondem a terapias convencionais ou que não aderem facilmente a determinado regime terapêutico. Como a hipertensão é um fator comum entre as DCs, os produtos naturais podem fornecer um espectro de tratamento totalmente novo para vários pacientes. Assim, gostaríamos de destacar a importância dos produtos naturais e talvez estimular a busca de novos agentes terapêuticos (naturais) que atuem como anti-hipertensivos, fornecendo embasamento científico para seu uso.
