# Supplementary material for: Pesquisando Compostos de Ocorrência Natural para Tratar Hipertensão
Source: Arq Bras Cardiol. 2021 Nov 22;117(6):1104–5. [Article in Portuguese] doi: 10.36660/abc.20210869 (PMC8757164; doi:10.36660/abc.20210869)
Supplement: Translation

# Translation: Searching Naturally Occurring Compounds to Treat Hypertension

**Authors:** Diego Santos Souza, Danilo Roman-Campos

**Affiliations:** 1 São Paulo SP Brazil Laboratório de CardioBiologia, Departamento de Biofísica, Escola Paulista de Medicina, Universidade Federal de São Paulo, São Paulo, SP – Brazil

**Keywords:** Cardiovascular Diseases, Hypertension, Diet, Biological Products, Mortality, Prevention and Control, Sauromatum Gluttatum/therapeutic use, Rats

Cardiovascular diseases (CDs) are the major cause of death worldwide. Impressively, approximately 17.9 million people died from CDs in 2019, representing almost 32% of global deaths. Also, nearly three-quarters of deaths due to CDs occur in low- and middle-income countries, which is a risk factor for Brazilians.^1^ There are several underlying mechanisms involved in CDs, and hypertension is a significant contributor. Today, it is thought that about 1.28 billion adults aged 30-79 years have hypertension in the world. Furthermore, it has been estimated that 60–70% of hypertension in adults is attributable to obesity, associated with insulin resistance, dyslipidemia and metabolic syndrome.^2^ Also, almost 50% of adults with hypertension are unaware of its condition, and only 20% have hypertension under control.^3^

According to the American College of Cardiology and the American Heart Association,^4^ Normal Blood pressure (BP) is considering when Systolic Blood Pressure (SBP) is <120 mm Hg and < 80 mm Hg for diastolic blood pressure (DBP). Elevated BP is considered when SBP is 120-129, and DBP is <80 mg. Stage 1 and 2 hypertension are considering when SBP is 130-139 mm Hg or > 140 mm Hg, respectively, or DBP is 80-89 mm Hg or >90 mm Hg, respectively. These values follow the most recent Brazilian guideline to diagnostic hypertension.^5^

The numbers show us that the search for new compounds to ameliorate hypertension is of great importance to treat patients worldwide. Repurposing therapies is one possibility. For example, a recent pilot study demonstrated that a single dose of infliximab could reduce mean and DBP levels immediately after its infusion, compared to placebo in patients with hypertension. However, not every patient may benefit from this type of therapy.^6^

In this scenario, natural products play an essential part. The implementation of natural products has increased over the last decades. In an in-depth review study that covered all-natural products as sources of new drugs, from 1981 to 2019 approved by the Food and Drug Administration, approximately 1946 new compounds were approved to treat human diseases. In the context of antihypertensive, 82 natural compounds were authorized.^7^ However, despite the increasing number of natural compounds authorized, it is still necessary to find new compounds to treat hypertension since patients are not responsive to a given antihypertensive class or do not adhere to the treatment.

Bibi et al.^8^ describe a novel crude plant extract from *Sauromatum guttatum* to treat experimental hypertension. In the manuscript, the authors evaluate the potential therapeutic application of the plant extract in the classical animal model of High salt-induced hypertension in rats (DOCA-hypertensive rats). Their study found that hypertensive animals daily treated for eight weeks with *S. guttatum* significantly attenuated Arterial Blood Pressure in a dose-dependent manner. Furthermore, 300 mg/kg of *S. guttatum* had a similar antihypertensive effect as Verapamil 15 mg/kg treated during the same time frame. The use of isolated aortic rings from hypertensive rats favored acetylcholine-dependent relaxation. Importantly, aortic Rings isolated from hypertensive rats orally treated with *S. guttatum* crude extract at 300 mg/kg for 28 days had a similar potency for acetylcholine-dependent relaxant effect against phenylephrine-induced contractions that observed for control. Interestingly, aortic rings isolated from hypertensive animals treated with Verapamil at 15 mg/kg for 28 days showed only modest improvement in the same experimental approach. Apparently, the mechanism involved in the relaxation effect of *S. guttatum* involves activating muscarinic receptors in endothelial cells and stimulating nitric oxide production. Also, the crude extract may interact with the L-type calcium channel found in aortic rings, which may also contribute to the antihypertensive effect observed. Thus, future studies should isolate the main antihypertensive active compound found in *S. guttatum* to uncover its potential further to treat hypertension in additional animal models in vivo.

Despite the growing number of natural products approved as therapeutic agents, considering hypertension, it is still necessary to find new compounds that act at low doses. Also, a natural compound could be helpful for compliance issues and patients who do not respond to a particular class of antihypertensive treatment. As hypertension is a common factor among CDs, natural products can provide a whole new treatment’ spectrum for several patients. Thus, we would like to highlight the importance of natural products and perhaps stimulate the search for new (natural) therapeutic agents that act as antihypertensive, providing a scientific basis for its use.
